# Forgiving Adolescents: Far From Depression, Close to Well-Being

**DOI:** 10.3389/fpsyg.2019.01725

**Published:** 2019-07-24

**Authors:** Barbara Barcaccia, Susanna Pallini, Andrea Pozza, Michela Milioni, Roberto Baiocco, Francesco Mancini, Giovanni Maria Vecchio

**Affiliations:** ^1^Department of Education, Roma Tre University, Rome, Italy; ^2^Associazione di Psicologia Cognitiva (APC) and Scuola di Psicoterapia Cognitiva srl (SPC), Rome, Italy; ^3^Department of Health Sciences, University of Florence, Florence, Italy; ^4^Department of Developmental and Social Psychology, Sapienza University of Rome, Rome, Italy; ^5^Department of Human Sciences, Guglielmo Marconi University, Rome, Italy

**Keywords:** depression, forgiveness, anger, adolescence, well-being

## Abstract

Forgiveness has been proven to be an effective way of regulating negative affect and decreasing depression. This study aimed at examining the relationship among constructs particularly relevant to adolescents’ well-being, including forgivingness (dispositional forgiveness) anger, depression and Hedonic Balance (HB). Specifically, using a structural equation modelling approach, the fully mediational role of the different facets of anger in the relationship between forgiveness and depression was tested in 773 adolescents, of which 69% girls. Results showed that forgivingness was positively and negatively related to, respectively, HB and depression, through a general effect of anger, suggesting that more forgiving adolescents had higher HB and lower depression as they reported a lower general tendency to experience anger. Forgivingness was also positively related both to HB and to depression through the mediation of all the facets of Anger. Moreover, only for HB, a specific effect of Anger-control was found, suggesting that more forgiving adolescents had higher HB as they reported higher strategies to control anger in a functional manner. The model invariance was supported across gender. Our results suggest that forgiveness is a significant protective factor against depression for adolescents, helping them to effectively control and manage anger, thus fostering emotional health. An important clinical implication of our study regards the potential of forgiveness as a resource for well-being in therapy: among the various possible protective factors in adolescence, forgiveness has the added advantage that it can be fostered in clinical settings, and working on forgiveness in psychotherapy or in counselling could decrease adolescent depression and improve well-being.

## Introduction

Adolescence is a critical period for the emergence of depressive symptoms, which can be viewed as a failure to accomplish the developmental task of regulating emotions (Cummings and Davies, 1996; Ahmed et al., 2015; Allen and Tan, 2016). This task is a particularly important one in light of the fact that adolescents have been found to experience more intense mood swings and emotional reactions to social stimuli than people of other ages, because of the hormonal changes associated with this developmental stage of life (Nelson et al., 2005). According to their ability to regulate emotions, they can have normal mood swings, or in some cases, they can experience moods and behaviours characterised by destructive anger and depression (Garnefski et al., 2005; Eysenck and Derakshan, 2011).

Regarding anger, Brunner and Spielberger (1979) consider it a multifaceted construct, implying several components. Trait Anger is a stable tendency to experience anger; State Anger corresponds to the intensity of angry feelings at a particular time; Anger Expression-Out implies the expression of angry feelings towards other persons or objects in the environment, whereas Anger Expression-In implies the maladaptive suppression of angry feelings. Anger encompasses also an adaptive component, Anger-Control, i.e., the capacity to control angry feelings by preventing the expression of anger or regulating angry feelings by calming down. In adolescence research, anger has been positively correlated to depression in both normal (Deffenbacher et al., 1996; Balsamo, 2010) and clinical populations (Fava and Rosenbaum, 1999; Koh et al., 2002). A strong association has been found between Anger Expression-Out/Anger Expression-In and depressive manifestations (Bridewell and Chang, 1997). Also, according to the Diagnostic and Statistical Manual of Mental Disorders, fifth edition (DSM-5) (American Psychiatric Association, 2013), a diagnosis of a major depressive episode “requires that a child or adolescent exhibits one of the two key features: depressed or *irritable mood* and a loss of interest or pleasure” (Goldstein and DeVries, 2017, p.153).

According to the attachment theory, the link between anger and depression seems to be related to the perception of having been hurt by another person, where one initially experiences anger, sometimes causing them to break the relation with that person, and subsequently experiences depression as a result of the ruptured relationship (Bowlby, 1979; Horwitz, 2004).

As damage to interpersonal relationships is related to anger and depression, the ability to repair relationships through forgiveness could be related to well-being and to the reduction of anger and depression (Akhtar and Barlow, 2016). Enright et al. (1998, pp. 47–48) defined forgiveness as “a willingness to abandon one’s right to resentment, negative judgement and indifferent behaviour towards one who has unjustly hurt us.” State-forgiveness (forgiving a specific offence) can be distinguished from trait-forgiveness, or *forgivingness*, an enduring tendency to forgive transgressions across situations and over time (Roberts, 1995). Across all ages, higher forgivingness is associated with lower levels of depression (Burnette et al., 2009) and anger (Fehr et al., 2010) and with higher levels of well-being (Toussaint and Webb, 2005).

Forgiveness has been proven to be an effective way of regulating negative affect (Worthington and Scherer, 2004; Barcaccia et al., 2018b). When people forgive, there is both a reduction of angry and resentful emotions, thoughts and behaviours, and an increase of the positive and benevolent ones towards the offending person (Wade et al., 2014). Affect regulation implies Hedonic Balance (HB), i.e., the balance between negative and positive emotions, the affective component of subjective well-being (Kahneman, 1999; Diener, 2000; Schimmack et al., 2002; Kahneman and Krueger, 2006).

The relationship between forgiveness, depression, and well-being is well-established (e.g., Toussaint and Webb, 2005; Burnette et al., 2009; Fehr et al., 2010). Increasing forgiveness leads to a reduction in depressive manifestations (e.g., Akhtar and Barlow, 2018). It has also been reported that promoting forgiveness increases Anger-Control and reduces Trait Anger and Anger Expression-Out/Anger Expression-In (Fitzgibbons, 1986; Gambaro, 2003; Harris et al., 2006; Wilkowski et al., 2010; Akhtar and Barlow, 2018).

There is a small number of studies investigating gender covariations in the relationship between forgivingness and psychological health outcomes (Miller and Worthington, 2015). According to meta-analyses (e.g., Fehr et al., 2010), females are typically more forgiving than males, whereas males are more vengeful than females.

Regarding gender differences in anger regulation strategies among adolescents, mixed results have been reported, with some studies showing no differences (e.g., Eschenbeck et al., 2007) and other studies showing females to have lower anger control strategies (Wong et al., 2018).

### Study Aims and Hypotheses

Therefore, it is important to investigate how one’s difficulty to forgive could be related to anger and depression: the inability to forgive increases anger, and persistent anger can, in turn, destroy interpersonal relationships, exposing one to feelings of failure and isolation, facilitating the onset of depressive symptoms. Based on these considerations, we intend to test a model that encompasses forgivingness, anger, depressive symptomatology and HB. While some authors have found an inverse relationship between forgivingness and depression (Burnette et al., 2009), forgivingness and anger (Watson et al., 2017), and between depression and anger (Balsamo, 2010), surprisingly no study has investigated, so far, the mediational role of anger in the relationship between forgivingness and depression.

Given that the inability to forgive is related to anger, and anger is related to depression, it is reasonable to posit that an unforgiving attitude is related to depression, with the mediational role of anger, and that forgivingness is related to HB, with the mediational role of anger control. To provide a more rigorous test of the hypothesised associations among the variables, we tested two alternative models as recommended by Quintana and Maxwell (1999), where depression/HB and anger facets, respectively, were considered as the independent variables.

It may be hypothesised that the inability to forgive others may increase Trait Anger and dysfunctional strategies like either expressing anger towards other persons or suppressing it.

On one hand, expressing anger towards other persons/objects or suppressing/holding anger in may lead to a range of cognitive, emotional and behavioural depressive responses like loneliness, hopelessness, feelings of guilt, low self-concept, self-blame and social withdrawal. On the other hand, the ability to forgive others may increase the capacity to control anger through functional cognitive, emotional and behavioural coping strategies; this may reduce depressive responses and increase HB by enhancing self-confidence and self-esteem, interpersonal adjustment and trust in others (Wai and Yip, 2009). Due to the small number of available studies and the mixed literature findings about gender covariations in the relationship between forgivingness and psychological health, we also tested whether the model was invariant across gender.

## Materials and Methods

### Participants and Procedure

The current study included 773 middle- and high-school students (69% females), ranging in age from 12 to 18 (*M*_*age*_ = 15.6 years, *SD* = 2.00). This study has been approved by the ethics committee of the Department of Developmental and Social Psychology, Sapienza University of Rome, and it has been performed in accordance with the ethical standards laid down in the 1964 Declaration of Helsinki and its later amendments. All the students completed the self-report questionnaires collectively during classes. Permission was obtained from the institutional school committees. In conformity with the Italian law, written informed consent was collected from students of legal age and from parents of underage students.

### Measures

The *Trait Forgivingness Scale* (TFS; Berry et al., 2005) assesses dispositional forgiveness. It contains 10 items (sample item: *I try to forgive others even when they don’t feel guilty for what they did*) to which participants report their agreement on a five-point Likert scale (1 = strongly disagree, 5 = strongly agree). The psychometric properties of the Italian TFS have been recently validated (Barcaccia et al., 2018a), suggesting a 7-item scale. Cronbach’s alpha for our study was 0.71.

The *State Trait Anger Expression Inventory-2 Child and Adolescent* (STAXI–2 C/A; Brunner and Spielberger, 1979) is a 35-item self-report scale. The State Anger Scale measures the intensity of angry feelings at a particular time (sample item: *I feel I am angry*), whereas the Trait Anger Scale measures individual disposition to experience anger as a personality characteristic (sample item: *I am a hot-headed person*). Anger Expression-Out measures the expression of anger toward other people or objects in the environment engaging in verbal or physically aggressive behaviours (sample item: *I show my anger*). Anger Expression-In measures the extent to which angry feelings are held in or suppressed (sample item: *I get mad inside, but do not show it*). Anger-Control assesses the ability to control the inward or outward expression of angry feelings (sample item: *I try to calm my angry feelings*). Participants respond using a 4-point scale, ranging from 1 (Hardly ever) to 3 (Often). Alpha coefficients ranged from 0.68 to 0.85 for the total and the subscales, similar to the original version (Brunner and Spielberger, 1979).

*Hedonic Balance* (HB) was assessed using the PANAS (Watson et al., 1988), a 20-item scale developed to measure two higher-order dimensions of self-rated positive and negative affect. The Positive Affect section includes terms such as “active,” “enthusiastic,” whereas the Negative Affect section includes terms such as “afraid,” “hostile.” Participants reported the intensity in which they have generally experienced each emotion on a 5-point scale, from 1 (never/not at all) to 5 (always/very much). Intensity of HB was evaluated by subtracting the negative affect score from the positive affect score. Intensity of HB is considered an indicator of subjective well-being, as suggested by findings on affect measurement and well-being (Schimmack et al., 2002).

The *Children’s Depression Inventory* (CDI; Kovacs, 1992) is a 27-item scale evaluating depressive symptoms in children and adolescents from 8 to 17 years old. Participants rate themselves based on how they feel and think, with each statement being identified with a rating from 0 to 2. Cronbach’s alpha for our sample was 0.84.

## Statistical Analyses

Preliminary correlational analyses were calculated through the SPSS 25 software. Correlation coefficients values were interpreted according to Cohen et al. (2014) as follows:0 < *r* < |0.30| = weak; |0.30| < *r* < |0.50| = moderate; |0.50| < *r* < |0.70| = strong; *r* > |0.70| = very strong.

### Structural Equation Analysis

In the present study, Mplus Version 8.1 (Muthén and Muthén, 1997–2017) was used to estimate the hypothesised model.

All variables were included in the model as single indicator latent variables by estimating the error terms from the reliability of the measures (Kline, 2015). The following indices were applied to evaluate the fit of the tested models: χ*2* statistic, Tucker and Lewis Index (TLI), Comparative Fit Index (CFI), and the Root Mean Square Error of Approximation (RMSEA). We accepted TLI and CFI values higher than 0.95 (Hu and Bentler, 1999) and RMSEA lower than 0.06 (Browne and Cudeck, 1993) as thresholds for good fit. We also used Akaike Information Criterion (AIC) to evaluate model fit, with lower values indicating a better fit (Kline, 2015). Burnham and Anderson (2004) suggested “as a rule of thumb” that models with *Δ*AIC*i* ≤ 2 have substantial support; models with 4 ≤ *Δ*AIC*i* ≤ 7 have considerably less support; models with *Δ*AIC*i* ≥ 10 have essentially no support.

In order to test the moderating effects of gender, we used multiple-group structural equation modelling (Muthén and Muthén, 2008). The equivalence between male and female groups was evaluated by imposing identical unstandardized estimates for paths (we refer to this model as the gender-constrained model). Finally, as suggested by Bollen (1989), we tested the plausibility of these equality constraints with the chi-squared difference test (*Δ*χ*^2^*) between nested models (i.e., the gender- constrained model vs. the unconstrained model).

### Mediation Analysis

In order to estimate mediation effects, we adhered to the procedures suggested by MacKinnon et al. (2004) using the Asymmetric Confidence Interval Method (ACI). We examined the hypothesised pattern of influences by estimating the following paths: (1) from forgivingness to Trait-Anger, Anger Expression-Out, Anger Expression-In and Anger Control; (2) from Trait-Anger, Anger Expression-Out, Anger Expression-In and Anger Control to HB; (3) from Trait-Anger, Anger Expression-Out, Anger Expression-In and Anger Control to depression. In addition, the four dimensions of anger were allowed to covary as were HB and depression.

## Results

### Descriptive and Correlational Analyses

Results of one-way ANOVA by gender are reported in Table 1.

**TABLE 1:** Means, standard deviations, and sex differences for forgivingness, anger, HB and depression for males and females.

	**Males**		**Females**			
**Variable**	**M**	**SD**	**M**	**SD**	***F*(1,773)**	***p***
1. Forgivingness	22.12	5.36	21.73	5.22	3.50	0.34
2. Trait Anger	18.27	3.72	19.65	3.67	22.91^*^	0.000
3. Anger Expression-Out	8.00	2.30	8.38	2.41	4.27^*^	0.039
4. Anger Expression-In	9.60	2.31	9.36	2.49	1.60	0.207
5. Anger Control	11.35	2.38	10.94	2.48	4.50^*^	0.034
6. Hedonic Balance	11.70	9.24	7.14	9.72	37.54^*^	0.000
7. Depression	39.74	7.07	45.24	6.98	101.56^*^	0.000

^*^p < 0.05. F = *F* ratio from one-way analyses of variance; within the parentheses are the degrees of freedom and the number of participants.

Correlational analyses are reported in Table 2. In both males and females Forgivingness is negatively correlated to Trait Anger and Expression anger-out and depression, positively with Anger control and HB. Trait anger and Anger Expression-out are negatively correlated with HB and positively with depression. Anger control is positively correlated with HB and negatively with depression.

**TABLE 2:** Zero-order correlations among measures of TFS, STAXI, HB, and CDI for males and females.

	**1**	**2**	**3**	**4**	**5**	**6**	**7**
1. Forgivingness	1	–0.31^∗∗^	–0.28^∗∗^	0.08	0.27^∗∗^	0.16^*^	–0.23^∗∗^
2. Trait Anger	–0.31^∗∗^	1	0.52^∗∗^	0.04	−0.14^*^	–0.26^∗∗^	0.26^∗∗^
3. Anger Expression-Out	–0.33^∗∗^	0.54^∗∗^	1	–0.34^∗∗^	–0.29^∗∗^	–0.26^∗∗^	0.18^∗∗^
4. Anger Expression-In	0.13^∗∗^	–0.04	–0.31^∗∗^	1	0.28^∗∗^	–0.03	0.06
5. Anger Control	0.19^∗∗^	–0.18^∗∗^	–0.35^∗∗^	0.22^∗∗^	1	0.21^∗∗^	–0.19^∗∗^
6. Hedonic Balance	0.17^∗∗^	–0.36^∗∗^	–0.27^∗∗^	–0.19^∗∗^	0.30^∗∗^	1	–0.46^∗∗^
7. Depression	–0.24^∗∗^	0.45^∗∗^	0.30^∗∗^	0.12^∗∗^	–0.16^∗∗^	–0.56^∗∗^	1

The correlation coefficients above the diagonal are for males; the correlation coefficients below the diagonal are for females. ^∗∗^*p* < 0.01; ^*^p < 0.05.

### Structural Equation Modelling

We preliminarily tested a model encompassing the path of gender and age on forgivingness and we found that the model did not show a good fit: χ*^2^*_(14)_ = 209.462, *p* < 0.001, CFI = 0.83, TLI = 0.58, RMSEA = 0.13 (95% CI:0.12–0.15), AIC = 30304.119. Thus, this model is not influenced by gender and age.

Subsequently, we tested a multi-group model (Table 3) encompassing both direct and indirect effects χ*^2^*_(0)_ = 0, *p* = 0.00, CFI = 1.00, TLI = 1.00, RMSEA = 0.00 (95% CI:0.00–0.00), AIC = 30129.38. Thus, the direct effect of forgivingness on depression and HB was not significant.

**TABLE 3:** Path coefficients of the full mediational model.

		**Dependent variable: Hedonic Balance (*Y*)**							
		***Direct effect* (*b*_i_)**							
**Independent variable**	**Mediators**	**Coefficient**		**SE**		***p***			
		***Males***	***Females***	***Males***	***Females***	***Males***	***Females***		
Forgivingness		−0.073	−0.068	0.043	0.040	0.091	0.091		
	
		***Specific indirect effects* (*a_i_b_i_*)**							
		**Coefficient**		***p***		**Lower**		**Upper**	
		***Males***	***Females***	***Males***	***Females***	***Males***	***Females***	***Males***	***Females***

Forgivingness	Trait-Anger	0.140	0.140	0.696	0.696	−0.450	−0.450	0.731	0.731
	Anger Expression-In	−0.182	−0.182	0.338	0.338	−0.495	−0.495	0.130	0.130
	Anger Expression-Out	0.063	0.063	0.913	0.913	−0.888	−0.888	1.014	1.014
	Anger Control	0.162	0.162	0.024	0.024	0.044	0.044	0.280	0.280
	
		***Total indirect effect* (*a_i_b_i_*)**							
	Anger facets	0.183	0.183	0.012	0.012	0.063	0.063	0.302	0.302

		**Dependent variable: Depression (*Y*)**							

		***Direct effect* (*b*_i_)**					
**Independent variable**	**Mediators**	**Coefficient**		**SE**		***p***			
		***Males***	***Females***	***Males***	***Females***	***Males***	***Females***		

Forgivingness		0.011	0.008	0.052	0.039	0.837	0.837		
	
		***Specific indirect effects* (*a_i_b_i_*)**							
		**Coefficient**		***p***		**Lower**		**Upper**	
		***Males***	***Females***	***Males***	***Females***	***Males***	***Females***	***Males***	***Females***

Forgivingness	Trait-Anger	−0.224	−0.224	0.584	0.584	−0.899	−0.899	0.450	0.450
	Anger Expression-In	0.056	0.056	0.801	0.801	−0.312	−0.312	0.425	0.425
	Anger Expression-Out	0.061	0.061	0.929	0.929	−1.068	−1.068	1.190	1.190
	Anger Control	−0.031	−0.031	0.651	0.651	−0.143	−0.143	0.081	0.081
	
		***Total indirect effect* (*a_i_b_i_*)**							
	Anger facets	−0.138	−0.138	0.019	0.019	−0.235	−0.235	−0.041	−0.041

The gender-unconstrained model without the direct effects showed a good fit: χ*^2^*_(4)_ = 3.569, *p* = 0.46, CFI = 1.00, TLI = 1.00, RMSEA = 0.00 (95% CI:0.000–0.073), AIC = 30124.950. We examined the gain in fit achieved by imposing equality constraints across genders. This model fitted the data well, χ*^2^*_(22)_ = 33.07, *p* = 0.06, CFI = 0.99, TLI = 0.98, RMSEA = 0.036 (95% CI:0.000–0.060), AIC = 30118.451. However, the change in fit between the gender-unconstrained versus the constrained model was not significant: *Δ*χ*^2^*_(18)_ = 29.500, *p* = 0.05, *Δ*CFI = 0.01 (Cheung and Rensvold, 2002).

We removed the equality constraints across genders in the correlation between Anger-Out and Anger Control, supporting the tenability of the constraints imposed across male and female groups.

**FIGURE 1:**
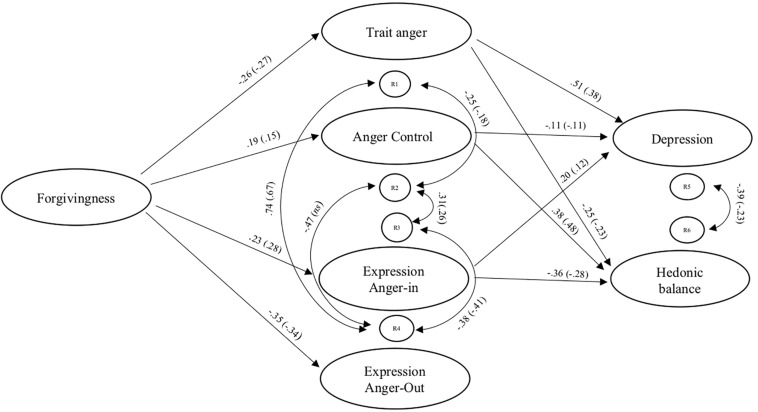
Standardised path coefficients for males and females (females in parentheses).

In Figure 1 we show the best fitting constrained model.

The anger facets were all related to each other, except for Trait-Anger with Expression Anger-In. HB and depression were also significantly and negatively related.

### Mediation Effect Modelling

The total indirect effect of the STAXI dimensions was statistically significant (the 95% asymmetric lower and upper CI limits did not include zero), thus supporting mediation of the conjoint effect of the STAXI subscales. A significant specific indirect effect was found only for Anger Control. Regarding depression, a significant total indirect effect of the STAXI dimensions was found, whereas indirect specific effects were not found (see Figure 1).

All the mediated paths were found to be equal for males and females. The model accounted for a similar proportion of variability for males and females. Specifically, for males, *R*-squared were 7% for Trait-anger, 4% for Anger Control, 5% for Expression Anger-In, 13% for Expression Anger-Out, 30% for depression and 34% for HB; for females, *R*-squared were 7% for Trait-anger, 2% for Anger Control, 8% for Expression Anger-In, 12% for Expression Anger-Out, 15% for depression and 34% for HB.

### Alternative Models

Two alternative models (AM) were tested. Since the two AMs were not nested in the hypothesised model, ΔAIC was used to compare their fit. In AM1, we considered depression and HB as independent variables, the four anger facets as mediators, and forgivingness as the outcome. This model showed acceptable fit: χ*^2^*(22) = 68.477, *p* < 0.001, CFI = 0.95, TLI = 0.91, RMSEA = 0.07 (95% CI:0.05–0.09), AIC = 30.153.858,; however, lower CFI and TLI values and higher RMSEA and AIC values than the hypothesised model (Δ AIC = 35.407) indicated that it was a worse approximation to the data. In this model, HB predicted all four anger facets, while depression predicted Trait-anger and Expression anger-out in males and Anger-control also in females; at the same time only Trait-anger predicted Forgivingness.

In AM2, we considered the anger facets as independent variables, forgivingness as the mediator, and depression and HB as the outcomes. The AM2 showed a worse fit than the hypothesised model as suggested by the fit indices: χ*^2^* (28) = 307.345, *p* < 0.001, CFI = 0.71, TLI = 0.57, RMSEA = 0.16 (95% CI:0.14–0.18), AIC = 30.380,726, and by a higher AIC (Δ AIC = 262,275). In this model, only the path linking Forgivingness to depression was statistically significant. For all the two AMs, the information deriving from the ΔAIC indicated that they had poor empirical support (ΔAIC ≥ 10) compared to the hypothesised mediational model.

## Discussion

The present study was the first investigation examining the relationship among forgivingness, anger, HB and depression in adolescents. Using a structural equation modelling approach, our results showed that forgivingness was positively and negatively related, respectively, to HB and depression, through a general effect of anger facets, suggesting that adolescents with higher forgivingness had higher HB and lower depression as they reported a lower general tendency towards anger. These results are consistent with previously reviewed literature highlighting the association between forgivingness and both depression (Brown, 2003; Burnette et al., 2009; Barcaccia et al., 2017, 2018b) and anger (Watson et al., 1988; Fehr et al., 2010; Barcaccia et al., 2018a). In addition, our findings expand on the existing literature in that they evidence a mediational model encompassing the relationship between forgivingness and HB and depression, with the general mediational effect of anger.

Another key-finding of our study was that forgivingness was positively related only to HB, through a specific effect of Anger-Control, suggesting that more forgiving adolescents had higher HB, as they had more functional strategies to control anger effectively. Thus, adolescents who have a higher ability to forgive others might report a higher subjective well-being (i.e., HB) because they have functional cognitive, emotional and behavioural strategies to regulate anger. This result was consistent with experimental data showing that increasing forgiveness leads to an increase in anger cognitive control skills, which in turn increases well-being (Wilkowski et al., 2010).

The lack of a mediational effect of all the other anger facets is, however, in contrast with our hypotheses; the present data also suggest that forgiveness might foster HB, specifically by increasing the ability of adolescents to regulate anger in a functional way, and not specifically by reducing Trait-Anger, or dysfunctional anger regulation strategies such as suppressing it or expressing it out towards other people/objects. Another result in contrast with our hypotheses was the lack of a direct effect of forgivingness on HB and depression. This evidence was not consistent with previous literature about the relationship between forgivingness and HB/depression suggesting that the capacity to forgive others may directly protect adolescents from depression and promote his/her wellbeing through a more functional mode of anger regulation.

We also found no evidence about the effect of gender in the model as the multigroup analysis supported gender invariance. This result is in line with those studies reporting no evidence of gender differences in the covariations between anger regulation and psychological health (e.g., Eschenbeck et al., 2007).

Our findings can be explained by hypothesising that dispositional forgiveness reduces the general effect of anger on HB helping individuals to refrain from negatively and depressively judging their inner experiences, such as emotions and thoughts (Barcaccia et al., 2019). Forgivingness may act as a protective factor against the detrimental effects of dysfunctional behaviours triggered by anger, such as aggressive acting-outs, out-bursts of anger (Paciello et al., 2017), severing ties with friends, and ruminating about others as dangerous, which in turn can lead to depressive feelings of loneliness and guilt regarding the disruption of interpersonal relations (Deffenbacher et al., 1996). Moreover, given the association between inflated responsibility beliefs and depression (Pozza and Dèttore, 2014), forgivingness may be hypothesised to reduce depression by attenuating adolescents’ inflated sense of duty/obligation/responsibility about the necessity of expressing vigorously one’s rage and frustration, thus helping adolescents to let go of anger rather than holding onto it. Consistent with previous research, the fact that forgivingness is inversely related to depression and directly to HB when anger is well regulated, suggests that dispositional forgiveness may include a set of emotional regulation skills such as emotional intelligence and perspective-taking (Rizkalla et al., 2008; Carvalho et al., 2010). The lack of an indirect effect of forgivingness on depression/HB through the other dysfunctional anger facets may suggest that the model could be improved by including other dysfunctional anger regulation strategies as mediators such as angry rumination, which has been found to be related to lower forgivingness and lower affect regulation (Berry et al., 2005). Future studies could include other dysfunctional strategies to cope with anger as mediators in the pathway from forgivingness to HB/depression.

Our findings offer several implications for the well-being of adolescents. First, school-based programs on forgiveness could promote a more benevolent attitude in confronting with slights and interpersonal ruptures, thereby preventing depression and increasing well-being. Such programs could promote functional regulation skills, such as emotional intelligence, empathy, and perspective taking (Onal and Yalcin, 2017); they can be easily delivered in group settings, making them particularly suitable for school contexts (Worthington et al., 2016). An important clinical implication of our study regards the potential of forgiveness as a resource for well-being in therapy: among the various possible protective factors in adolescence, forgiveness has the added advantage that it can be fostered in clinical settings, and working on forgiveness in psychotherapy or in counselling could decrease adolescent depression and improve well-being.

Our study presents some limitations, firstly regarding the use of self-reported measures, and secondly because of the cross-sectional nature of our data. Nevertheless, regarding the first limitation, it must be noted that forgivingness, anger, and depression are private cognitive-emotive states that are necessarily accessible through report by the individuals who hold these beliefs and experience these emotional tonalities. However, it is certainly true that future studies could strengthen the investigation of these topics by relying upon multiple methods and informants across different situations, to minimise possible biases due to self-reporting and reputation. Regarding the cross-sectional nature of this study, future research could also investigate whether forgivingness is a protective factor for the onset of depression from a longitudinal perspective. On this basis, educational programs aimed at increasing adolescents’ capacity to forgive by productively processing their feelings of anger, could act as preventive strategies for depression. In fact, forgivingness education programs have been proven effective in increasing forgivingness and decreasing anger and depression (e.g., Enright et al., 2007). Our findings are based on a large sample of Italian adolescents, thus future studies could explore these dimensions also in other cultural contexts.

Obviously, depression has more facets than those considered in this study: one cannot exclude the possibility that other personal or situational factors moderate or mediate the relationship between forgivingness and depression. Future studies could also take into account the quality of the interpersonal relationships in which the offences occurred, and to what extent the individual feels helpless and impotent in the face of such offences, given the debate on the importance of helplessness and hopelessness in both the genesis and the maintenance of depression (Bastounis et al., 2016).

Overall, these results offer notable indications for mental health professionals, as well as for educators: helping young people to forgive is not to be considered strictly as a facet of moral education, as it can represent a useful contribution in enhancing emotional and psychological health in adolescents, preventing the onset of depression by helping them to effectively manage anger. Forgivingness implies a balanced consideration of oneself and the other, and the consequent balance is not solely cognitive, but mainly emotional. In this perspective, emotional regulation and a balanced consideration of self and others appear to be two sides of the same coin.

## Data Availability

The datasets generated for this study are available on request to the corresponding author.

## Ethics Statement

This study has been approved by the Ethics Committee of the Department of Developmental and Social Psychology, Sapienza University of Rome. and all procedures have been performed in accordance with the ethical standards laid down in the 1964 Declaration of Helsinki and its later amendments, and in conformity with the Italian law. We gathered written informed consent from all participants. All the students completed the paper–pencil self-report questionnaires collectively during classes. Permission was obtained from the institutional school committees. In conformity with the Italian law, informed consent was collected from students of legal age and from parents of underage students. Pupils were assured that participation was optional and that their responses would remain anonymous.

## Author Contributions

BB had the initial idea, collected data, and drafted the manuscript. SP gave substantial contribution to the design of the work and revised it critically for its intellectual content. AP contributed to the conception and design of the study and assisted in the statistical analysis. MM assisted in the statistical analyses. RB organised the database. FM revised the work. GV performed the statistical analyses and co-wrote the first draft of the manuscript. BB, SP, AP, MM, RB, and GV wrote sections of the manuscript. All authors contributed to manuscript revision, read and approved the submitted version.

## Conflict of Interest Statement

The authors declare that the research was conducted in the absence of any commercial or financial relationships that could be construed as a potential conflict of interest.

## References

[B1] Ahmed S. P. Bittencourt-Hewitt A. Sebastian C. L. (2015). Neurocognitive bases of emotion regulation development in adolescence. *Dev. Cogn. Neurosci.* 15 11–25. 10.1016/j.dcn.2015.07.006 26340451 PMC6989808

[B2] Akhtar S. Barlow J. (2016). Forgiveness therapy for the promotion of mental well-being. a systematic review and meta-analysis. *Trauma Violence Abuse* 19 1–17. 10.1177/1524838016637079 27009829

[B3] Akhtar S. Barlow J. (2018). Forgiveness therapy for the promotion of mental well-being: a systematic review and meta-analysis. *Trauma Violence Abuse* 19 107–122. 10.1177/1524838016637079 27009829

[B4] Allen J. P. Tan J. (2016). “The multiple facets of attachment in adolescence,” in *Handbook of Attachment: Theory, Research, and Clinical Applications*, 3rd Edn eds Cassidy J. Shaver P. R. (New York, NY: Guilford Press), 399–415.

[B5] American Psychiatric Association (2013). *Diagnostic and Statistical Manual of Mental Disorders*, 5th Edn Arlington, VA: American Psychiatric Press 10.1176/appi.books.9780890425596

[B6] Balsamo M. (2010). Anger and depression: evidence of a possible mediating role for rumination. *Psychol. Rep.* 106 3–12. 10.2466/PR0.106.1.3-12 20402420

[B7] Barcaccia B. Baiocco R. Pozza A. Pallini S. Mancini F. Salvati M. (2019). The more you judge the worse you feel. A judgemental attitude towards one’s inner experience predicts depression and anxiety. *Pers. Individ. Diff.* 138 33–39. 10.1016/j.paid.2018.09.012

[B8] Barcaccia B. Milioni M. Pallini S. Vecchio G. M. (2018a). Resentment or forgiveness? The assessment of forgivingness in Italian adolescents. *Child Indic. Res.* 11 1407–1423. 10.1007/s12187-017-9483-6

[B9] Barcaccia B. Pallini S. Baiocco R. Salvati M. Saliani A. M. Schneider B. (2018b). Forgiveness and friendship protect adolescent victims of bullying from emotional maladjustment. *Psicothema* 30 427–433. 10.7334/psicothema2018.11 30353845

[B10] Barcaccia B. Schneider B. H. Pallini S. Baiocco R. (2017). Bullying and the detrimental role of un-forgiveness in adolescents’ wellbeing. *Psicothema* 29 217–222. 10.7334/psicothema2016.251 28438245

[B11] Bastounis A. Callaghan P. Banerjee A. Michail M. (2016). The effectiveness of the Penn Resiliency Programme (PRP) and its adapted versions in reducing depression and anxiety and improving explanatory style: a systematic review and meta-analysis. *J. Adolesc.* 52 37–48. 10.1016/j.adolescence.2016.07.004 27494740

[B12] Berry J. W. Worthington E. L. O’Connor L. E. Parrott L. Wade N. G. (2005). Forgivingness, vengeful rumination, and affective traits. *J. Pers.* 73 183–226. 10.1111/j.1467-6494.2004.00308.x 15660677

[B13] Bollen K. A. (1989). A new incremental fit index for general structural equation models. *Sociol. Methods Res.* 17 303–316. 10.1177/0049124189017003004

[B14] Bowlby J. (1979). *The Making and Breaking of Affectional Bonds.* London: Tavistock Publications.

[B15] Bridewell W. B. Chang E. C. (1997). Distinguishing between anxiety, depression, and hostility: relations to anger-in, anger-out, and anger control. *Pers. Individ. Diff.* 22 587–590. 10.1016/S0191-8869(96)00224-3

[B16] Brown R. P. (2003). Measuring individual differences in the tendency to forgive: construct validity and links with depression. *Pers. Soc. Psychol. Bull.* 29 759–771. 10.1177/0146167203029006008 15189631

[B17] Browne M. W. Cudeck R. (1993). “Alternative ways of assessing model fit,” in *Testing Structural Equation Models.* eds Bollen K.A. Long J.S. Newbury Park, CA: Sage.

[B18] Brunner T. M. Spielberger C. D. (1979). *State-Trait Anger Expression Inventory-2 Child and Adolescent (Staxi-2 C/A).* Lutz, FL: PAR.

[B19] Burnette J. L. Davis D. E. Green J. D. Worthington E. L. Bradfield E. (2009). Insecure attachment and depressive symptoms: the mediating role of rumination, empathy, and forgiveness. *Pers. Individ. Dif.* 46 276–280. 10.1016/j.paid.2008.10.016

[B20] Burnham K. P. Anderson D. R. (2004). Multi-model inference understanding AIC and BIC in model selection. *Sociol. Methods Res.* 33 261–304. 10.1177/0049124104268644

[B21] Carvalho D. Neto F. Mavroveli S. (2010). Trait emotional intelligence and disposition for forgiveness. *Psychol. Rep.* 107 526–534. 10.2466/02.09.20.21.PR0.107.5.526-534 21117479

[B22] Cheung G. W. Rensvold R. B. (2002). Evaluating goodness-of-fit indexes for testing measurement invariance. *Struct. Equ. Modeling* 9 233–255. 10.1097/NNR.0b013e3182544750 22551991 PMC3361901

[B23] Cohen P. West S. G. Aiken L. S. (2014). *Applied Multiple Regression/Correlation Analysis for the Behavioral Sciences.* New York, NY: Routledge.

[B24] Cummings E. M. Davies P. (1996). Emotional security as a regulatory process in normal development and the development of psychopathology. *Dev. Psychopathol.* 8 123–139. 10.1017/S0954579400007008

[B25] Deffenbacher J. L. Oetting E. R. Lynch R. S. Morris C. D. (1996). The expression of anger and its consequences. *Behav. Res. Ther.* 34 575–590. 10.1016/0005-7967(96)00018-16 8826765

[B26] Diener E. (2000). Subjective well-being: the science of happiness and a proposal for a national index. *Am. Psychol.* 55 34–43. 10.1037/0003-066X.55.1.34 11392863

[B27] Enright R. D. Freedman S. Rique J. (1998). “The psychology of interpersonal forgiveness” in *Exploring Forgiveness.* eds Enright R. D. North J. (Madison, WI: University of Wisconsin press), 46–62.

[B28] Enright R. D. Knutson J. A. Holter A. C. Baskin T. Knutson C. (2007). Waging peace through forgiveness in Belfast, Northern Ireland II: educational programs for mental health improvement of children. *J. Res. Educ.* 17 63–78.

[B29] Eschenbeck H. Kohlmann C. W. Lohaus A. (2007). Gender differences in coping strategies in children and adolescents. *J. Individ. Diff.* 28 18–26. 10.1027/1614-0001.28.1.18

[B30] Eysenck M. W. Derakshan N. (2011). New perspectives in attentional control theory. *Pers. Individ. Diff.* 50 955–960. 10.1016/j.paid.2010.08.019 22364371

[B31] Fava M. Rosenbaum J. F. (1999). Anger attacks in patients with depression. *J. Clin. Psychiatry* 60 21–24. 10418810

[B32] Fehr R. Gelfand M. J. Nag M. (2010). The road to forgiveness: a meta-analytic synthesis of its situational and dispositional correlates. *Psychol. Bulletin.* 136 894–914. 10.1037/a0019993 20804242

[B33] Fitzgibbons R. P. (1986). The cognitive and emotive uses of forgiveness in the treatment of anger. *Psychother. Theor. Res. Pract. Train.* 23 629–633. 10.1037/h0085667

[B34] Gambaro M. E. (2003). *School-Based Forgiveness Education in the Management of Trait Anger in Early Adolescents.* Doctoral dissertation University of Wisconsin, Madison, WI.

[B35] Garnefski N. Kraaij V. van Etten M. (2005). Specificity of relations between adolescents’ cognitive emotion regulation strategies and internalizing and externalizing psychopathology. *J. Adolesc.* 28 619–631. 10.1016/j.adolescence.2004.12.009 16203199

[B36] Goldstein S. DeVries M. (eds). (2017). *Handbook of DSM-5 Disorders in Children and Adolescents.* Cham: Springer International Publishing 10.1007/978-3-319-57196-6

[B37] Harris A. H. Luskin F. Norman S. B. Standard S. Bruning J. Evans S. (2006). Effects of a group forgiveness intervention on forgiveness, perceived stress, and trait-anger. *J. Clin. Psychol.* 62 715–733. 10.1002/jclp.20264 16538652

[B38] Horwitz L. (2004). The capacity to forgive: intrapsychic and developmental perspectives. *J. Am. Psychoanal. Assoc.* 53 485–511. 10.1177/00030651050530021401 16045162

[B39] Hu L. T. Bentler P. M. (1999). Cut-off criteria for fit indexes in covariance structure analysis: conventional criteria versus new alternatives. *Struct. Equ. Modeling* 6 1–55. 10.1080/10705519909540118

[B40] Kahneman D. (1999). “Objective happiness,” in *Well-being: The Foundations of Hedonic Psychology*, eds Kahneman D. Diener E. Schwarz N. (New York, NY: Russell Sage Foundation), 1–23.

[B41] Kahneman D. Krueger A. B. (2006). Developments in the measurement of subjective well-being. *J. Econ. Perspect.* 20 3–24.

[B42] Kline R. B. (2015). *Principles and Practice of Structural Equation Modeling.* New York, NY: Guilford Publications.

[B43] Koh K. B. Kim C. H. Park J. K. (2002). Predominance of anger in depressive disorders compared with anxiety disorders and somatoform disorders. *J. Clin. Psychiatry* 63 486–490. 10.4088/JCP.v63n0604 12088159

[B44] Kovacs M. (1992). *Children’s Depression Inventory.* North Tonawanda, NY: Multi-Health System.

[B45] MacKinnon D. P. Lockwood C. M. Williams J. (2004). Confidence limits for the indirect effect: distribution of the product and resampling methods. *Multivariate Behav. Res.* 39 99–128. 10.1207/s15327906mbr3901_4 20157642 PMC2821115

[B46] Miller A. J. Worthington E. L. (2015). “Sex, forgiveness, and health,” in *Forgiveness and Health*, eds Toussaint L. Worthington E. Williams D. R. (Dordrecht: Springer), 10.1007/978-94-017-9993-5

[B47] Muthén L.K. Muthén B.O. (1997–2017). *Mplus User’s Guide.* Los Angeles, CA: Muthén & Muthén.

[B48] Muthén L. K. Muthén B. O. (2008). *Mplus (Version 5.1).* Los Angeles, CA: Muthén & Muthén.

[B49] Nelson E. E. Leibenluft E. McClure E. B. Pine D. S. (2005). The social re-orientation of adolescence: a neuroscience perspective on the process and its relation to psychopathology. *Psychol. Med.* 35 163–174. 10.1017/S003329170400391 15841674

[B50] Onal A. A. Yalcin I. (2017). Forgiveness of Others and Self-Forgiveness: the predictive role of cognitive distortions, empathy, and rumination. *Eurasian J. Educ. Res.* 68 97–120.

[B51] Paciello M. Muratori P. Ruglioni L. Milone A. Buonanno C. Capo R. (2017). Personal values and moral disengagement promote aggressive and rule breaking behaviours in adolescents with disruptive behaviour disorders: a pilot study. *Int. J. Offender Ther. Comp. Criminol.* 61 46–63. 26138350 10.1177/0306624X15589593

[B52] Pozza A. Dèttore D. (2014). Are inflated responsibility beliefs specific to OCD? Meta-analysis of the relations of responsibility to OCD, anxiety disorders, and depression symptoms. *Clin. Neuropsychiatry* 11 170–181.

[B53] Quintana S. M. Maxwell S. E. (1999). Implications of recent developments in structural equation modeling for counseling psychology. *Couns. Psychol.* 27 485–527.

[B54] Rizkalla L. Wertheim E. H. Hodgson L. K. (2008). The roles of emotion management and perspective taking in individuals’ conflict management styles and disposition to forgive. *J. Res. Pers.* 42 1594–1601.

[B55] Roberts R. C. (1995). Forgivingness. *Am. Philos. Q.* 32 289–306.

[B56] Schimmack U. Radhakrishnan P. Oishi S. Dzokoto V. Ahadi S. (2002). Culture, personality, and subjective well-being: integrating process models of life satisfaction. *J. Pers. Soc. Psychol.* 82:582. 10.1037/0022-3514.82.4.582 11999925

[B57] Toussaint L. Webb J. R. (2005). “Theoretical and empirical connections between forgiveness, mental health, and well-being,” in *Handbook of Forgiveness*, ed. Worthington E. L. Jr. (New York, NY: Routledge), 349–362.

[B58] Wade N. G. Hoyt W. T. Kidwell J. E. Worthington E. L. Jr. (2014). Efficacy of psychotherapeutic interventions to promote forgiveness: a meta-analysis. *J. Consult. Clin. Psychol.* 82 154–170. 10.1037/a0035268 24364794

[B59] Wai S. T. Yip T. H. J. (2009). Relationship among dispositional forgiveness of others, interpersonal adjustment and psychological well-being: implication for interpersonal theory of depression. *Pers. Individ. Diff.* 46 365–368. 10.1016/j.paid.2008.11.001

[B60] Watson D. Clark L. A. Tellegen A. (1988). Development and validation of brief measures of positive and negative affect: the PANAS scales. *J. Pers. Soc. Psychol.* 54 1063–1070. 10.1037/0022-3514.54.6.1063 3397865

[B61] Watson H. Rapee R. Todorov N. (2017). Forgiveness reduces anger in a school bullying context. *J. Interpers. Violence* 32 1642–1657. 10.1177/0886260515589931 26101439

[B62] Wilkowski B. M. Robinson M. D. Troop-Gordon W. (2010). How does cognitive control reduce anger and aggression? The role of conflict monitoring and forgiveness processes. *J. Pers. Soc. Psychol.* 98 830–840. 10.1037/a0018962 20438227

[B63] Wong T. K. Konishi C. Zhao K. (2018). Anger and anger regulation among adolescents: a consideration of sex and age differences. *Can. J. Behav. Sci.* 50 1–8. 10.1037/cbs0000089

[B64] Worthington E. L. Griffin B. J. Lavelock C. R. Hughes C. M. Greer C. L. Sandage S. J. (2016). “Interventions to promote forgiveness are exemplars of positive clinical psychology,” in *The Wiley Handbook of Positive Clinical Psychology*, eds Wood A. M. Johnson J. (Hoboken, NJ: John Wiley & Sons), 365–380.

[B65] Worthington E. L. Scherer M. (2004). Forgiveness is an emotion-focused coping strategy that can reduce health risks and promote health resilience: theory, review, and hypotheses. *Psychol. Health* 19 385–405. 10.1080/0887044042000196674

